# Correlation between electrical characteristics and biomarkers in breast cancer cells

**DOI:** 10.1038/s41598-021-93793-6

**Published:** 2021-07-12

**Authors:** Yang Wang, Ying Li, Jie Huang, Yan Zhang, Ren Ma, Shunqi Zhang, Tao Yin, Shangmei Liu, Yan Song, Zhipeng Liu

**Affiliations:** 1 Institute of Biomedical Engineering, Chinese Academy of Medical Sciences and Peking Union Medical College, Tianjin, 300192 China; 2 National Cancer Center/National Clinical Research Center for Cancer/Cancer Hospital, Chinese Academy of Medical Sciences and Peking Union Medical College, Beijing, 100021 China; 3 Dept of Mechanical Engineering, University College London, London, UK; 4 School of Physics, Taishan University, Taian, 271000 China

**Keywords:** Biophysics, Cancer

## Abstract

Both electrical properties and biomarkers of biological tissues can be used to distinguish between normal and diseased tissues, and the correlations between them are critical for clinical applications of conductivity (σ) and permittivity (ε); however, these correlations remain unknown. This study aimed to investigate potential correlations between electrical characteristics and biomarkers of breast cancer cells (BCC). Changes in σ and ε of different components in suspensions of normal cells and BCC were analyzed in the range of 200 kHz–5 MHz. Pearson's correlation coefficient heatmap was used to investigate the correlation between σ and ε of the cell suspensions at different stages and biomarkers of cell growth and microenvironment. σ and ε of the cell suspensions closely resembled those of tissues. Further, the correlations between Na^+^/H^+^ exchanger 1 and ε and σ of cell suspensions were extremely significant among all biomarkers (p_ε_ < 0.001; p_σ_ < 0.001). There were significant positive correlations between cell proliferation biomarkers and ε and σ of cell suspensions (p_ε/σ_ < 0.05). The microenvironment may be crucial in the testing of cellular electrical properties. ε and σ are potential parameters to characterize the development of breast cancer.

## Introduction

With the discovery of marked differences between the electrical impedances of malignant breast tissues and healthy tissues^1,2^, bioelectrical impedance technology has emerged as a new approach for breast cancer detection. This technology is noninvasive, cost-effective, safe, informative, and easily acceptable to both doctors and patients^3^. The conductivity (σ) and permittivity (ε) of different biological tissues not only vary in terms of frequency, but also change with their physiological and pathological states; therefore, these can be used as indicators for biomedical or clinical applications to detect and diagnose diseases^4^. Although certain frequency ranges of electrical properties have been linked to pathological states of tissues, reports correlating σ and ε with physiological and pathological characteristics of cells are rare^5^. However, for realizing clinical applications of σ and ε, it is critical to understand these correlations.

Tissue physiological status, such as the statuses of cellular activities^6^, membrane proteins^7^, and extra-cellular matrix^8^, have been reported to correlate with electrical characteristics. Wang et al. found that σ of liver cancer tissues increased in the frequency range of 10 Hz to 100 MHz as cell activity decreased^6^, and that the changes in σ and ε were more sensitive under 1 MHz. Weyer et al. found that overactivation of ion channel protein on human embryonic kidney (HEK) 293 cell membrane leads to increased extracellular σ^9^. The electrical properties of breast cancer were found that changes in the number of proteins on the cell membrane of breast cancer cells at different stages had no significant effect on σ^10^. Meanwhile, breast cancer cytoskeleton studies have found that increasing the density of the cytoskeleton leaded to decreased σ^11^. They sought to investigate the effects of breast cancer cell structure and membrane protein quantity of σ. Breast cancer has unique characteristics of tumors, such as unlimited proliferation, abnormal energy metabolism and acidic microenvironment^12^. These are the main pathological features that distinguish tumors from normal tissues. However, they did not investigate the correlation between the pathological changes in these tumorigenesis processes and electrical properties.

In order to reflect the changes of electrical properties caused by pathological changes of cells, the measurement methods of cell σ are mainly divided into single cell and cell suspension. The results of single cell electrical properties of breast cancer showed that electrical properties can distinguish different stages of breast cancer. In order to overcome the measurement error of electrical characteristics caused by different growth cycles of single cells, cell suspension resuspended with isotonic solution was used^13^. The electrical characteristics of breast cancer cell suspension showed that σ of MDA-MB-231 breast cancer cell was lower than that of normal breast cancer cell MCF-10A, abnormally. Although σ error of the cell suspension resuspended with isotonic solution is reduced, the measurement of electrical properties at the cellular level remains a challenge. Because the electrical properties that are measured in tissue come not only from the cell but also from the microenvironment that the cell is in^14^. However, the electrical properties measurement in cell level does not take into account of the influence on σ by the cell microenvironment, while the material exchange with the microenvironment is inevitable during the cell growth process.

Therefore, in this study, we aimed to establish a method to measure σ and ε of breast cancer cells with tumor microenvironment, and find the correlation between cell σ and biomarkers expressed during breast cancer development. The electrical characteristics of normal and breast cancer cells with different degrees of malignancy were detected between 200 kHz and 5 MHz. The changes in σ and ε of different components in cell suspension were analyzed and their correlations with biochemical parameters (biomarkers) of breast cancer cells were systematically evaluated using biology heatmap, which aims to provide biological basis for the clinical application of the potential diagnostic method based on electrical characteristics.

## Results

### Conductivity and permittivity of breast cells in 200 kHz–5 MHz

**Figure 1:**
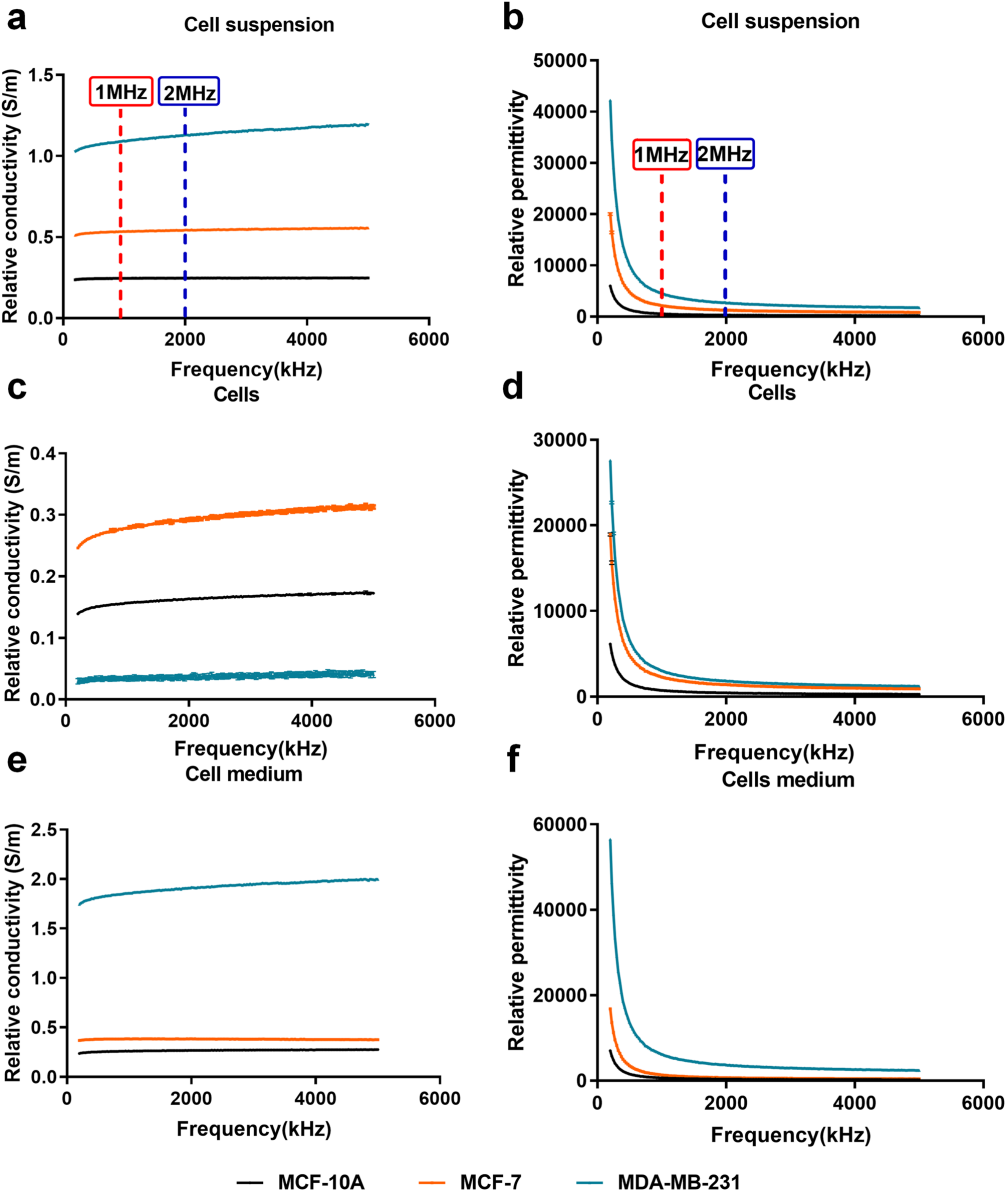
σ and ε of normal glandular mammary epithelial cell MCF-10A, breast cancer cell MCF-7, and MDA-MB-231 in the frequency range 200 kHz–5 MHz. Variation trend of σ and ε in cell suspensions group (**a**,**b**), cells group (**c**,**d**) and cell medium group (**e**,**f**). (Data are means ± SD, n = 10).

Experimental conditions are crucial for the impedance measurements. In addition to controlling the measured temperature, we measured the activity of the cells within 2.5 h (Fig. S1). It was found that there was no significant change in cell activity during this period. The effect of the radio frequency (RF) we used on the cell suspension system was further examined. Cell activity results showed that radiofrequency reduced cell activity, but not significantly (Fig. S2). RF also had no significant effect on the acidity of the medium (Fig. S3). On this basis, σ and ε of the breast cell lines with low metastasis (MCF-7) and high metastasis (MDA-MB-231) were measured in the frequency range of 200 kHz to 5 MHz and compared with those of the normal glandular mammary epithelial cell line (MCF-10A; Fig. 1).

The σ of each cell suspension increased slightly when the frequency was between 200 kHz and 1 MHz, and plateaued for frequencies beyond 1 MHz (Fig. 1a). The σ of MCF-10A cells was the lowest in the whole frequency range, while σ of MCF-7 cells with low metastasis was higher than that of MCF-10A, but much lower than that of the highly metastatic MDA-MB-23l breast cancer cells, indicating that cell σ was positively correlated to metastasis of breast cancer cells. The ε of all cell lines rapidly decreased with increasing frequency from 200 kHz to 2 MHz, and gradually levelled off at frequencies above 2 MHz (Fig. 1b). In particular, the differences in ε among the two breast cancer cells and normal cells decreased remarkably. Moreover, the ε of the three types of breast cells in suspensions were consistent with those for the σ. The normal breast cell suspension had the lowest ε and the breast cancer cell suspension with the highest metastasis had the highest ε.

The σ and ε of the breast cells group were shown in Fig. 1c,d. Compared with the cell suspensions group, the MDA-MB-231 cells group showed lowest σ and the MCF-7 cells showed the highest σ (Fig. 1c). The trend of ε in the cells group was consistent with that in breast cell suspensions (Fig. 1d). However, the difference between the ε of the MDA-MB-231 and MCF-7 cells group was smaller than that in their cell suspensions.

The σ and ε of the cell media group were also measured (Fig. 1e,f). The results were consistent with those of the breast cells suspension group. The σ and ε of MDA-MB-231 cells were much higher than those of MCF-7 cells, particularly in terms of the magnitudes compared to those in the cell suspensions group.

### Conductivity and permittivity of breast cells at 1 MHz

**Figure 2:**
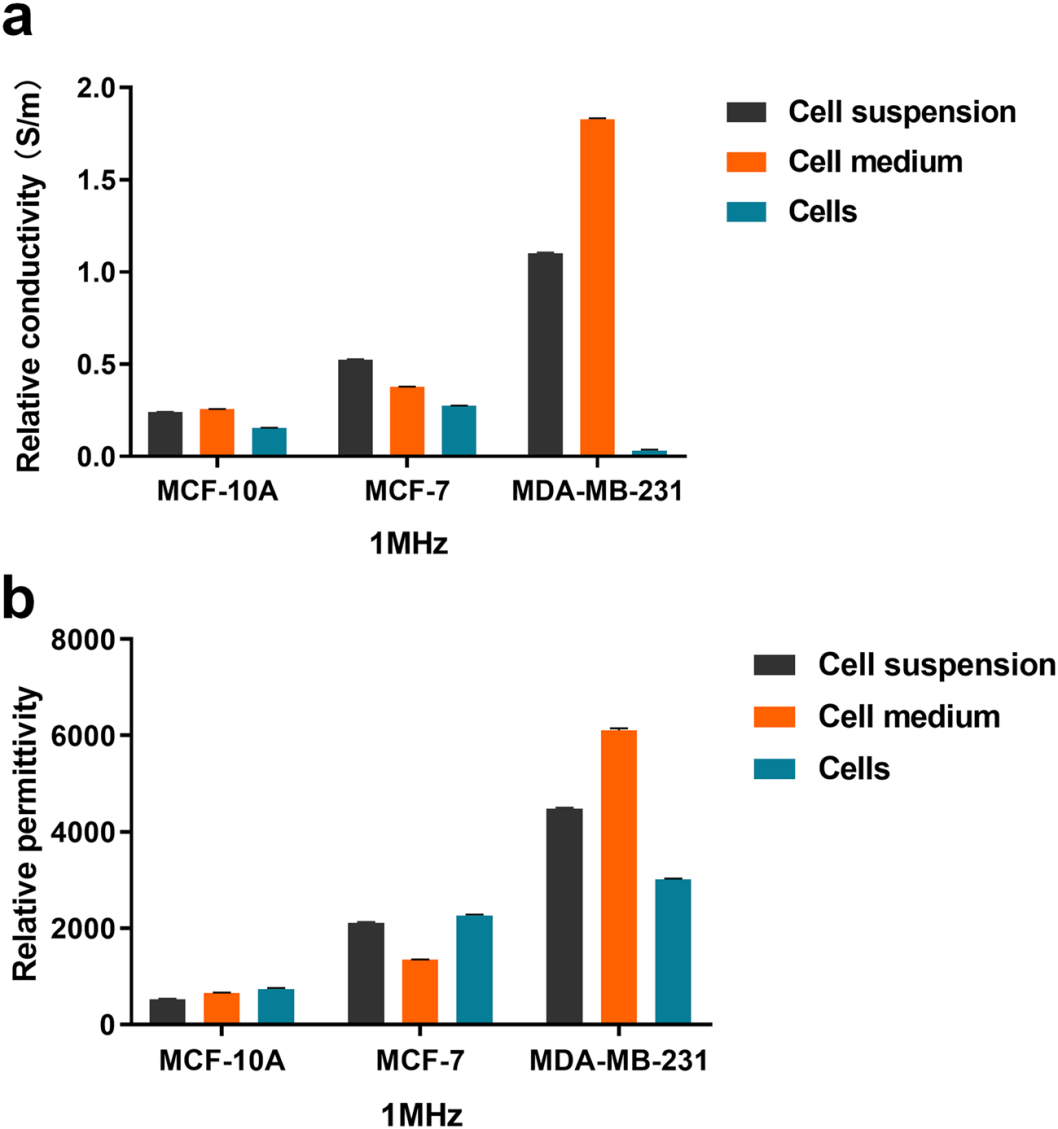
Comparison among the σ and ε of the three types of cells, cell media, and cell suspensions at 1 MHz. (**a**) Comparison among σ. (**b**) Comparison among ε. (Data are means ± SD, n = 10).

The σ and ε of the three groups were compared and analyzed at 1 MHz (Fig. 2)^6^. The trend of σ and ε mentioned above can be clearly observed at the different cell stages. Looking at the σ or ε of three groups of the same cell species, we found that the data of the cell suspension group were more comprehensive. The cell suspensions group was more representative as a whole. In addition, the σ of the cell media group were higher than those of the cells group at 1 MHz; this was particularly evident in the MDA-MB-231 cell lines (Fig. 2a), in which σ were more similar to those of the cell suspensions group, demonstrating the influence of culture medium.

### Migration and expression of Ki67 and cyclinD1

**Figure 3:**
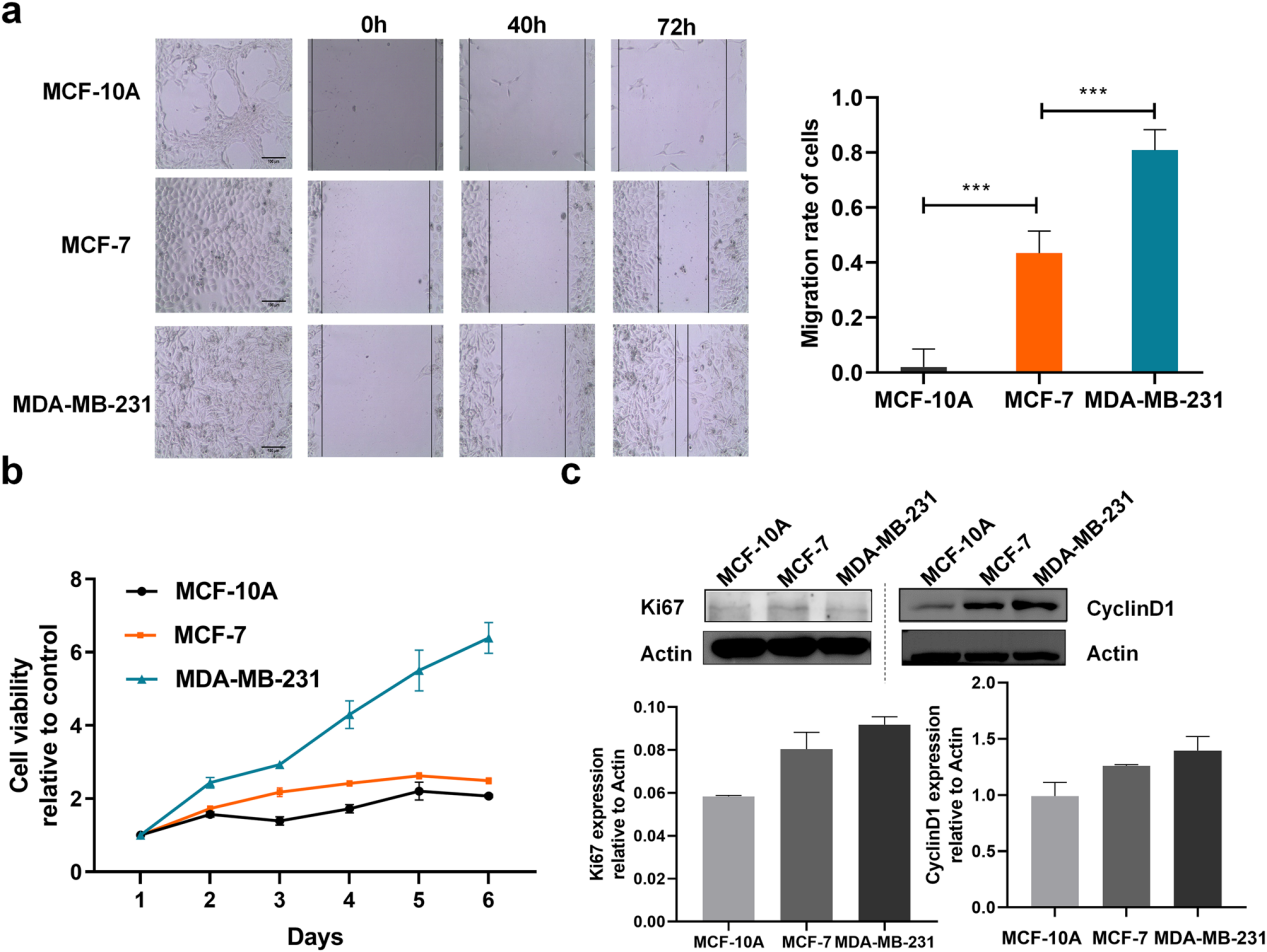
Migration and expression of Ki67 and cyclinD1 in three types of breast cells. (**a**) Micrographs of the migration of three types of breast cells (MCF-10A, MCF-7 and MDA-MB-231) during 72 h culture. Comparison of the migration rate in terms of area coverage calculated using Image J, as shown in the right. (Data are means ± SD, n = 6). (**b**) The growth curves of the three types of breast cells during 6 days of culture. (Data are means ± SD, n = 3). (**c**) Western blot of Ki67 and cyclinD1. The expression levels of Ki67, cyclinD1 in comparison with actin control for three cell types were quantified using Image J and presented below. (Data are means ± SEM, n = 3). *p < 0.05; **p < 0.01; **p < 0.001.

The migration rate of breast cancer cells with different degrees of malignancy during 72 h culture was compared with that of normal breast cells (Fig. 3a). The migration rate of MDA-MB-231 cells, which had the highest transference rate, was the fastest and the gap was reduced rapidly. The migration rate was slower for MCF-7 cells, but much faster than that of MCF-10A cells. Normal mammary MCF-10A cells were almost nonmigratory during the 72 h of culture.

The cell proliferation rates of the three types of breast cells were also measured using CCK-8 assay (Fig. 3b). MDA-MB-231 cells had the highest growth rate during the 6 days of culture. Similar to the cell migration study, the proliferation rates of breast cells were correlated with and accelerated by the increase in malignancy.

The expression of the cell proliferation protein Ki67 and cell cyclin protein cyclinD1 were shown in Fig. 3c. The degree of protein expression was quantified in comparison to the actin control; the expression levels of both Ki67 and cyclinD1 increased with increase in cell malignancy. These results were consistent with those of cell proliferation and migration rate.

### Changes in lactic acid and NHE1 levels in the acidic microenvironment

**Figure 4:**
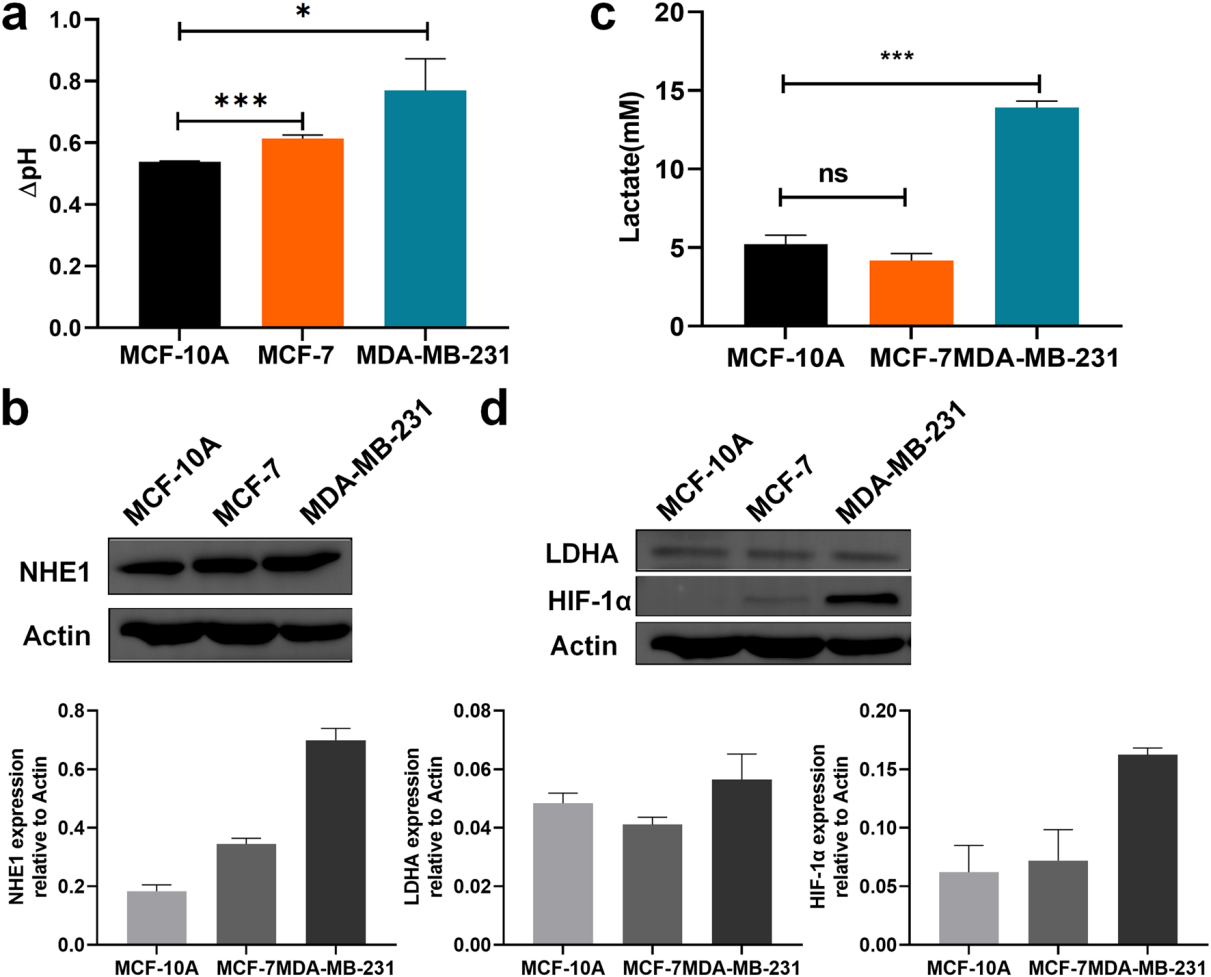
Changes in lactic acid and NHE1 levels in the acidic microenvironment of three types of cells. (**a**) Decrease in pH of culture media for 3 types of breast cells. (**b**) Western blot of NHE1. (**c**) The amount of lactic acid secreted in the three types of cell media. (**d**) Western blot of LDHA and HIF-1α. (Data are means ± SEM, n = 3). *p < 0.05; **p < 0.01; **p < 0.001.

As shown in Fig. 2, the conductivities and dielectric properties of the cultured medium contribute remarkably to these of the cell suspension Tumor microenvironments are typically acidic. In this study, changes in the culture media pH for three types of breast cells during culture were measured; the decrease in pH was shown in Fig. 4a. The pH values of the three culture mediums decreased as malignancy increased.

The expression level of Na^+^/H^+^ exchanger 1 (NHE1), which transports H^+^ to the extracellular space, was determined by Western blot assay (Fig. 4b). The degree of NHE1 protein expression in comparison with actin control was quantified. The expression level of NHE1 increased with increase in cell malignancy, thus explaining the decrease of pH in the culture media.

Abnormal glucose metabolism in tumor cells affects the acidic tumor microenvironment. Tumor cells undergo aerobic glycolysis to produce large amounts of lactic acid, which may affect the electrical properties of the cell suspension. The lactic acid levels in the three types of breast cell cultures were compared (Fig. 4c). There was no statistical difference in lactate output between normal MCF-10A and MCF-7 cells. Lactate excretion in MDA-MB-231 cells with high transferability was significantly higher than that in the other two types of cells. The expression of lactate dehydrogenase A (LDHA)^15^ was shown in Fig. 4d. LDHA expression was consistent with the lactic acid measurement; its expression was lower in MCF-7 cells than in normal MCF-10A cells, although there was no significant difference between the two groups. LDHA expression is regulated by hypoxia-inducible factor alpha (HIF-1α)^16,17^. The expression of HIF-1α was very low in MCF-10A cells, and the level of expression increased as the degree of malignancy of the breast cancer cells increased.

### Correlations between the conductivity and permittivity of breast cells and biomarkers

**Figure 5:**
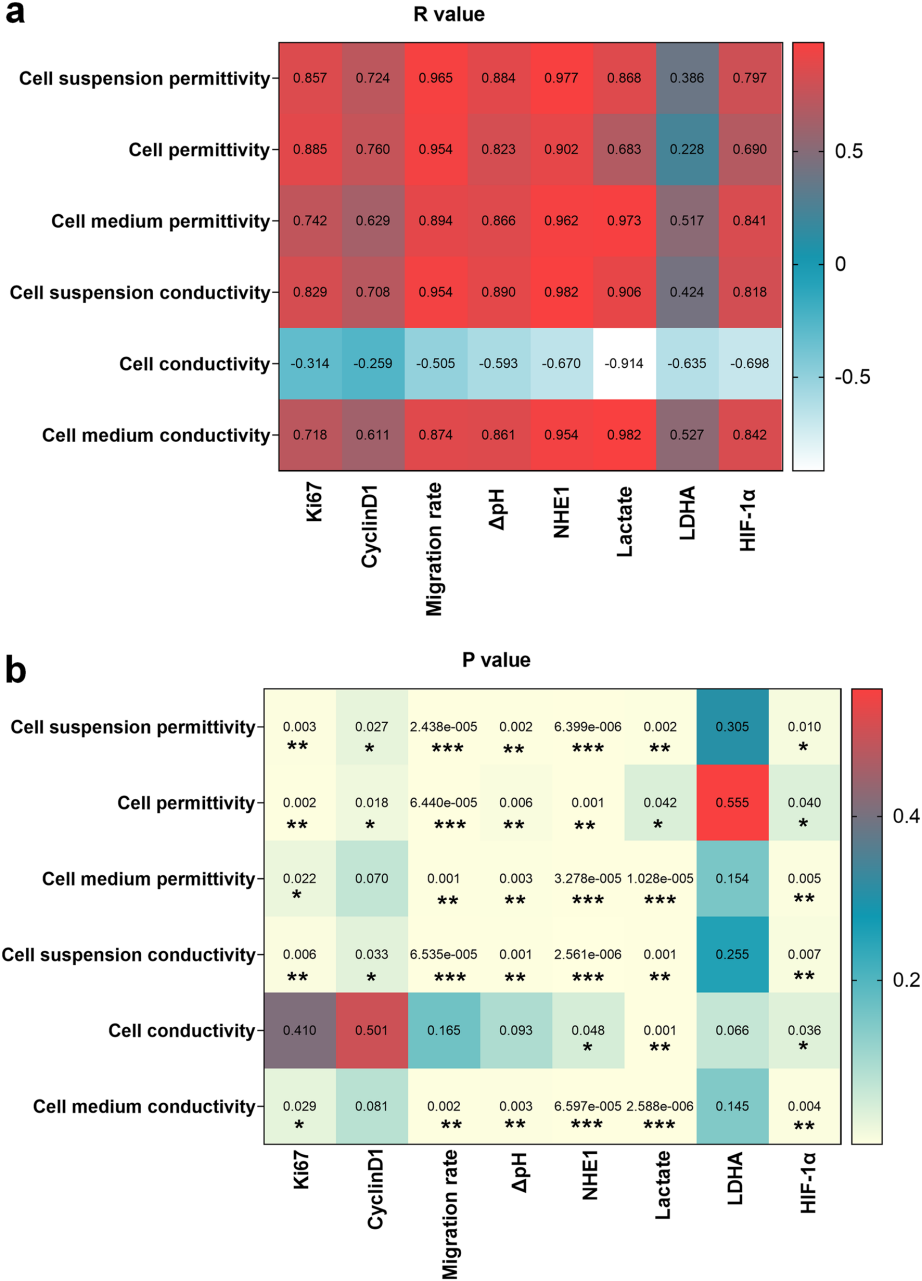
R and p values between the electrical characteristics of breast cells and biomarkers at 1 MHz. (**a**) Heatmap of r values. (**b**) Heatmap of p values.

To understand the relationship between the biological markers expressed by the three groups of breast cells and their σ and ε, we generated biological heatmaps of Pearson correlation coefficient, including the correlations between migration rate, pH change, lactate production, and expression of Ki67, cyclinD1, NHE1, LDHA, HIF-1α, and σ and ε of cell suspensions, cells, and culture medium at 1 MHz (Fig. 5). The most significant correlation between σ and cell suspension was the expression of NHE1 (p_σ_ < 0.001). The significant correlation between σ of cell medium was the production of lactic acid and the expression of NHE1 (p_σ_ < 0.01, p_σ_ < 0.01, respectively). Figure 5 clearly showed a negative correlation between cell σ and the levels of lactic acid, HIF-1α, and NHE1 (p_σ_ < 0.05).

Figure 5 showed that the expression of NHE1 is significantly correlated with the ε of cell suspension (p_ε_ < 0.001). The most significant correlation between the ε of cell culture media was lactic acid metabolism regulated by HIF-1α (p_ε_ < 0.001). The migration rate of cell lines was significantly correlated with the dielectric properties of cells (p_ε_ < 0.001).

In cell suspensions, the correlation between the σ and ε and NHE1 was more significant than those with other biomarkers.

## Discussion

The technology of bioelectrical impedance has attracted much interest owing to its potential for use in disease detection. Bioimpedance analysis show that the electrical properties of normal tissue, surrounding tissues, and carcinoma are different. Understanding the electrical behavior of mammary tissue and cells will aid the development of a noninvasive technique for early breast cancer detection. However, there is limited understanding of the correlation between the electrical characteristics of breast cancer tissues and the clinical pathological observations or the biological biomarkers used in clinical practice. In this study, the correlations between σ and ε and several biomarkers, including those involved in cell proliferation and microenvironment of cell growth, were investigated using cells at different stages of breast cancer development. Our results showed that the σ and ε of breast cancer cell suspension were consistent with those of breast cancer tissue^18^. Moreover, the changes in σ and ε during breast cancer development were closely related to the biomarkers of cell proliferation and cell growth microenvironment. The understanding obtained through this study will help apply bioelectrical characteristics in the development of breast cancer diagnosis tools.

σ and ε of breast cancer cells in the current study were consistent with previously reported results. Due to enlarged nuclear folding and increased membrane permeability in MDA-MB-231 cells, the σ of MCF-7 was higher than that of normal breast cells (MCF-10A), while the σ of MDA-MB-231, which had the highest metastasis, was the lowest^10^. However, this was inconsistent with the σ of breast cancer tissues^19^. Understandably, the characteristics of breast cancer tissues cannot be completely simulated by the suspended breast cancer cells in an isotonic salt solution, and the electrical characteristics of breast cancer cells cannot fully represent those of breast cancer tissues. In addition to the breast cancer cells, the living environment for cells in breast cancer tissues exert influence. As expected, σ and ε of breast cancer cell suspensions, which increased with increase in the degree of breast cancer malignancy, were consistent with the electrical characteristics of breast cancer tissues reported previously^18^. These results suggested that the σ and ε of breast cancer tissues may be closely reflected by the interaction of the cells that constitute them and their cellular microenvironments.

To verify our hypothesis, σ and ε of the cell culture media were analyzed. Results showed that the σ and ε of the cell culture media were consistent with those of the cell suspensions and breast cancer tissues. Since the biomolecules, materials, and energy are continuously exchanged between cells and their own microenvironment^20^, the microenvironment for cell survival cannot be ignored when analyzing the electrical characteristics of tissues at the cellular level. In fact, the σ of the cell culture solution contributed significantly to the overall σ. Therefore, study on the electrical characteristics of breast cancer cell suspension should be preferably considered as a whole, instead of cells alone, as it exhibited greater resemblance with breast cancer tissue. At the same time, the results have shown that the σ of cell suspensions, cells and cell media was significantly correlated with acidic microenvironment biomarkers. Therefore, the cell microenvironment cannot be excluded in the measurement of tissue σ, and also, the microenvironment must be taken into account on the establishment of cell model.

σ and ε can be effectively used to distinguish normal tissue from tumor tissue, although there is no systematic study on the correlation between σ and ε and tumor biological characteristics at present. Qiao et al.^10^ found that changes in the number of proteins on the membranes of breast cancer cells at different stages had no significant effect on σ. However, Weyer et al.^9^ found that overactivation of ion channel protein on human embryonic kidney 293 cell membrane led to an increase in extracellular σ. This suggests that changes in the number of proteins on the membranes are probably not associated with σ. However, ion channel proteins are related to the change of σ. In addition, a major factor affecting σ is electrolytes. And hence, the acidic microenvironment which may be a kind of tumor’s electrolytes is a major feature of tumors. Therefore, we speculated that physiological activity markers that may cause changes in hydrogen ion concentration in tumors are related to electrical properties. Therefore, we analyzed the correlation of biomarkers with σ and ε in some characteristics of tumor cells, including proliferation (KI67, cyclinD1), migration, acidic microenvironment (pH, NHE1), hypoxia microenvironment (HIF-1α), and abnormal energy metabolism (LDHA).

**Figure 6:**
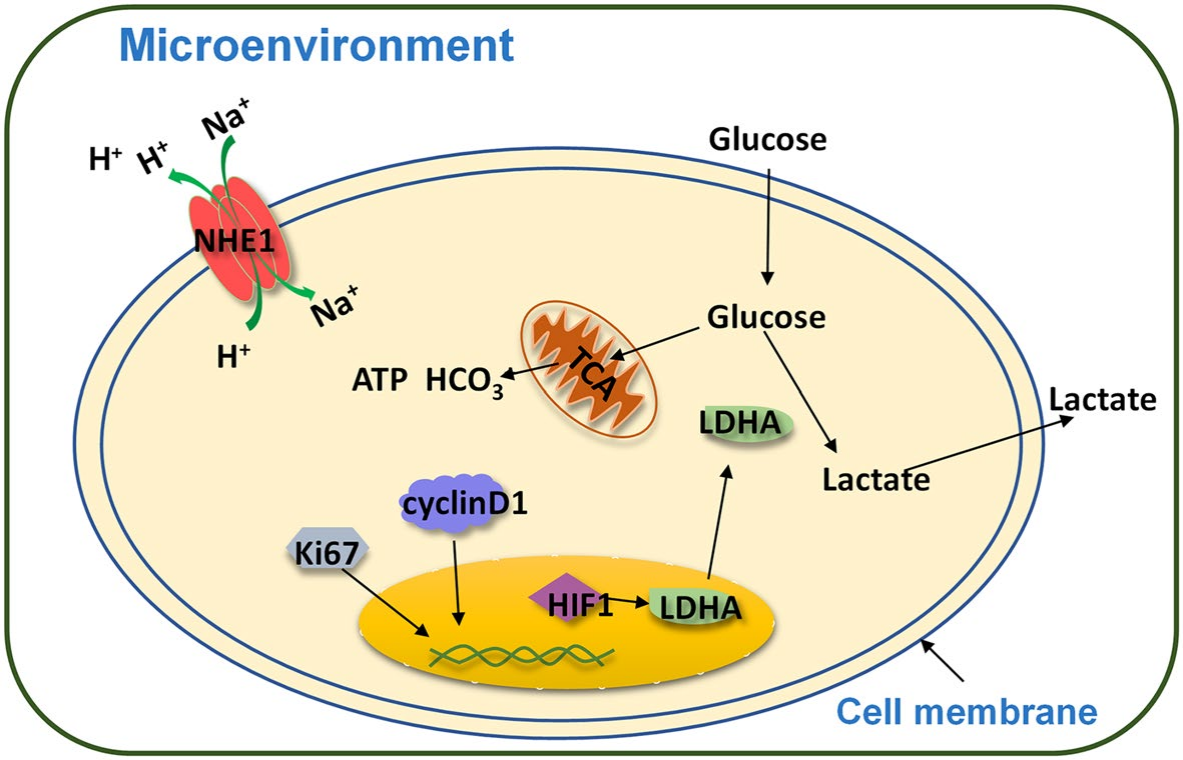
Schematic diagram of proteins promoting tumor development.

The most basic characteristic of tumors is their indefinite proliferation^12^. Ki67 is a cell proliferation antigen, which has been recognized as an early diagnostic indicator^21^ in the standard pathological assessment of breast cancer. CyclinD1 is a G1/S phase specific and highly conserved cyclin, and its expression rate has been used to reflect the degree of tumor malignancy^22,23^. The rapid proliferation and migration of breast cancer cells require a large amount of energy^24,25^, resulting in a large accumulation of H^+^ and lactic acid in MDA-MB-231 cells^26,27^ (Fig. 6). This process will greatly increase the concentration of H^+^ in the cell. This may alter the electrical properties of the cell. At the same time, monocarboxylate transporter 4 (MCT4) is responsible for the transport of lactic acid and H^+^^28^, but the large accumulation of lactic acid makes MCT4 unable to undertake the transport of H^+^^29^. In order to maintain intracellular acid homeostasis, NHE1 is overexpressed and becomes the main hydrogen ion transporter in breast cancer cells^30–33^. We also observed a significant increase in NHE1 expression in MDA-MB-231 cells. Further, after H^+^ transport occurs in breast cancer, the concentration of H^+^ in the cell decreases and reverts to basicity. As a result, the concentration of H^+^ outside the cell goes up. This process may alter the electrical properties of the cell's microenvironment. It has been reported that extracellular acidity is reduced after NHE1 expression is inhibited in breast cancer MCF-7 and MDA-MB-231 cells^34^. NHE1 becomes a key protein in changing H^+^ concentration inside and outside breast cancer cells. Overall, the increase of cell suspension σ together with the increase of malignant degree of breast cancer cells may be related to the large production and continuous transport of H^+^, and NHE1 may play a key role in the change of cell suspension σ. Consequently, NHE1 exhibited an extremely significant positive correlation with σ and ε of the cell suspension. σ and ε showed significant positive correlation with the above-mentioned proteins, which promote the development of breast cancer cells.

In this study, by comparing the electrical properties of different cell suspension components, it was found that the trend of σ and ε in cell suspension was consistent with those of biological tissue in the frequency range of 200 kHz–5 MHz. Further, the characteristics of cells and microenvironment were analyzed, and σ and ε of the cell suspension were found to have the most significant correlation with NHE1 among all the markers. Among the markers of microenvironment, the ionic marker NHE1 had the strongest correlation with σ and ε. Among the cell growth markers, the correlation between migration rate and σ and ε was the most significant. This indicated that the influence of microenvironment on σ and ε of a cell suspension cannot be ignored. However, 2D cell culture limits the cell density, making it impossible to structurally mimic the tissue more closely. Thus, future work should focus on studying the correlation between electrical properties and biomarkers in animal models using minimally invasive carrier electrodes. Collectively, the significant positive correlations between electrical properties and biomarkers proved that σ and ε are important indicators for the occurrence and development of breast cancer.

## Materials and methods

### Cell culture

Human breast cell lines MCF-10A, MCF-7, and MDA-MB-231 were purchased from Cell Resource Center, Institute of Basic Medical Sciences, Chinese Academy of Medical Sciences/Peking Union Medical College (Beijing; China). MCF-10A is a normal glandular mammary epithelial cell line, while MCF-7 and MDA-MB-231 are breast cancer cell lines with low and high degrees of division, respectively. MCF-10A and MCF-7 cells were cultured in Roswell Park Memorial Institute 1640 medium (RPMI 1640; Gibco; USA) and MDA-MB-231 cells were maintained in Dulbecco's modified Eagle's medium (DMEM; Gibco). Both cultures were supplemented with 10% fetal bovine serum (FBS; BI), 100 units/mL penicillin, and 100 μg/mL streptomycin. Cells were cultured in 5% CO_2_ humidified incubator (Thermo; Germany) at 37 ℃.

To prepare cell samples, 8 mL cell suspensions (10^5^ cells/mL) were plated on a 10 cm culture dish and incubated at 37 ℃. After 72 h, the cells were digested with trypsin and centrifuged at 500*g* for 5 min at room temperature. The supernatants were collected and used as suspension media.

### Detection of electrical characteristics

In this study, there were three groups with different types of samples: (A) cells group, in which three breast cell lines were collected in phosphate-buffered saline solution (PBS; Gibco)^35^; (B) cell suspensions group, in which the same breast cells were resuspended in their supernatants; (C) cell medium group, consisting of the supernatants collected from the cultures of three breast cell lines.

The dielectric properties of the three sample groups were measured using a liquid measuring device (16452A; KEYSIGHT; USA) connected to an electrical impedance analyzer (E4990A; KEYSIGHT).

The equipment was fully calibrated for air, with open circuit to short circuit settings. The initial air calibration range was 34.9 pF ± 25%; after careful control of humidity and air compositions, the test system was stabilized in the range of 32 pF ± 1 pF. The calibration was performed and adjusted to the acceptable range before each measurement. The measurement chamber was sterilized using 75% ethanol before testing.

The cell suspensions in each test were carefully shaken to ensure uniform distribution. A continuous liquid intake method was adopted to prevent the cells from settling to the bottom. Ten complete electrical impedance measurements were obtained from 3.8 mL cell samples, with frequencies varying from 200 kHz to 5 MHz; there were 195 frequency points with linear intervals.

The relative dielectric constants and dielectric losses of cell samples were obtained from the measurement directly. Then, σ of cells was calculated using the following relationship:

$$\upsigma = \upomega {\varepsilon _0}\varepsilon _r^{\prime \prime}$$

where σ is conductivity of the sample, ω is the angular frequency, and $\varepsilon _r^{\prime \prime}$ is the dielectric loss. ${\varepsilon}_{0}$ is the dielectric parameter of vacuum.

### Cell migration assay

Cells were seeded in 6-well plates at a density of 5 × 10^5^ cells/well. When approaching full confluence, six intersecting lines were scratched with 10 μL pipette tips. After replaced by the serum-free medium, images were first taken at 4 h and then every 8 h for two consecutive days using an optical microscope. Then, the areas and distances between the separated cells were measured at each time point using Image J.

### Cell proliferation assay

Breast cells were seeded into a 96-well plate at a density of 2000 cells/well and the cell proliferation was measured using Cell Counting Kit-8 (CCK8) cell viability kit every 24 h for 5 days. At each time point, fresh CCK8 medium was added to the 96-well plate according to the manufacturer’s instruction and the cells were incubated at 37 ℃ for 2 h. When absorbance was measured at 450 nm, the amount of formazan produced was directly proportional to the cell number.

### Extracellular pH detection

Next, 8 mL cell suspensions were seeded in culture dishes at a density of 10^5^ cells/mL and incubated in 5% CO_2_ humidified incubator at 37 ℃ for 3 days. The culture medium was used as the test control and 3 mL supernatant culture solution was injected into the measurement chamber at room temperature (25 ℃ ± 1 ℃). The pH meter (METTLER TOLEDO; Switzerland) was calibrated using pH calibration solutions (pH of 4.01, 7.00, or 9.01). The pH values of the samples were recorded accordingly.

### Extracellular lactic acid assay

The lactate production levels were determined using a Lactate Assay kit (Biovision, Milpitas, CA, USA). The cells were cultured in a 6-cm culture dish at a density of 10^6^ cells/dish at 37 ℃ in a 5% CO_2_ humidified incubator. After 24 h, the culture medium was replaced with serum-free DMEM/1640 medium and then incubated for 8 h. The cell sample was centrifuged at 500*g* for 5 min to obtain the culture medium supernatant. The lactic acid content was measured following the manufacturer’s instruction. The optical density (OD) at 570 nm was measured using Varioskan flash (Thermo; USA).

### Immunoblotting

Western blotting was used to analyze the proliferation (Ki67, cyclinD1) and microenvironment biomarkers (Na^+^/H^+^ exchanger 1, NHE1; lactate dehydrogenase A, LDHA; hypoxia-inducible factor alpha, HIF-1α) of three breast cells.

Cell lysate (Beyotime; China) with protease inhibitor (Biosharp; China) was added to the collected cells, which were fully lysed at 4 ℃ for 30 min. These were centrifuged for 10 min at 8000*g* at 4 ℃. The bicinchoninic acid (BCA; TIANGEN; China) method was used to determine the protein concentration. After all samples were adjusted to the same concentration, 1/4 v/v dodecyl sulfate, sodium salt-polyacrylamide gel electrophoresis (SDS-PAGE) sample loading buffer (Beyotime) was added to denature the samples for 10 min at 95 ℃. The proteins were separated by SDS-PAGE gel electrophoresis (Bio-Rad; USA) and transferred to polyvinylidene fluoride membrane (PVDF; Millipore; USA) by wet method.

To display proteins of different sizes on a single membrane, the membranes were cut prior to antibody hybridization. The membranes were incubated overnight at 4℃ with primary antibodies, including Anti-actin (CST; 3700T), Anti-Ki67 (Affinity Biosciences; AF0198), Anti-cyclinD1 (abcam; ab16663), Anti-NHE1 (abcam; ab67314), Anti-LDHA (abcam; ab101562), and Anti-HIF-1α (abcam; ab1). After washing with TBST three times for 10 min each time, horseradish peroxidase (HRP) conjugated secondary antibody (abcam) was added and incubated for 1 h at room temperature. Then, the film was covered with enhanced fluorescent color solution (Tanon; China) for development.

### Statistical analyses

All data were processed and analyzed using the SPSS software (IBM Corp; Armonk; NY) and GraphPad Prism 7 (GraphPad Software; USA). The means and standard deviations were obtained. Using ImageJ software, western blot grayscale and the cell area from scratch in the cell migration measurement were determined. A student’s t test was used to compare the differences between the two groups.

## Supplementary Information

Supplementary Information 1.
