# Supplementary material for: Correlation between electrical characteristics and biomarkers in breast cancer cells
Source: Sci Rep. 2021 Jul 12;11:14294. doi: 10.1038/s41598-021-93793-6 (PMC8275571; doi:10.1038/s41598-021-93793-6)
Supplement: Supplementary file 1 — Supplementary Information 1. [file 41598_2021_93793_MOESM1_ESM.pdf]

Correlation between electrical characteristics and biomarkers in breast cancer cells

Yang Wang[1], Ying Li[1], Jie Huang[3], Yan Zhang[4], Ren Ma[1], Shunqi Zhang[1], Tao Yin[1], Shangmei Liu[2], Yan Song[2, *], Zhipeng Liu[1, *]

[1]Institute of Biomedical Engineering, Chinese Academy of Medical Sciences and Peking Union Medical College, Tianjin 300192, China.

[2]Department of Pathology, National Cancer Center/National Clinical Research Center for Cancer/Cancer Hospital, Chinese Academy of Medical Sciences, Beijing 100021, China

[3]Dept of Mechanical Engineering, University College London, London, UK

[4]School of Physics, Taishan University, Taian, 271000, China

*** Correspondence:**
Corresponding Author： Yan Song, Zhipeng Liu
songyan@cicams.ac.cn, lzpeng67@163.com

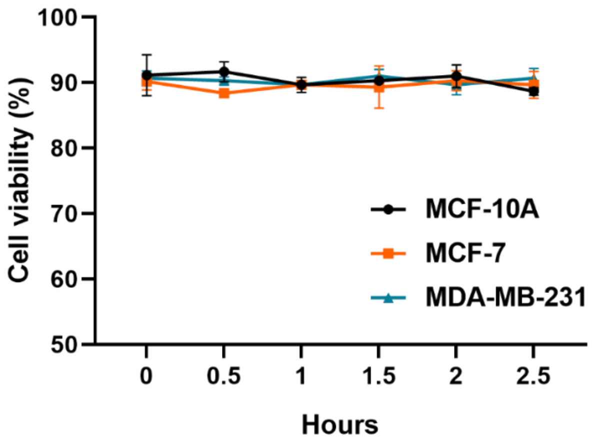

Fig. S1 Cell viability changes within2.5 h. (Data are means ± SD, n=3).

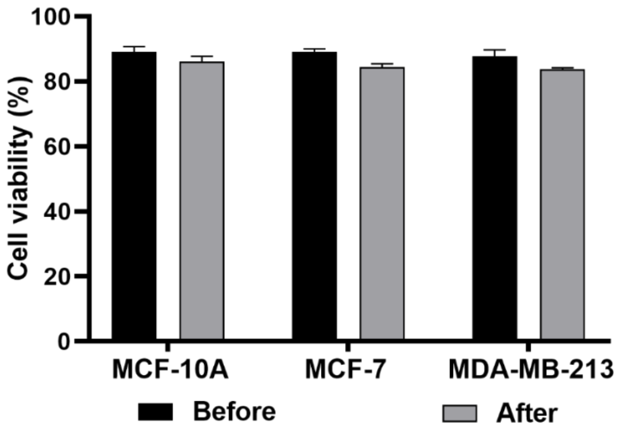

Fig. S2. Cell viability changes before and after impedance measurements. (Data are means ± SD, n=3).

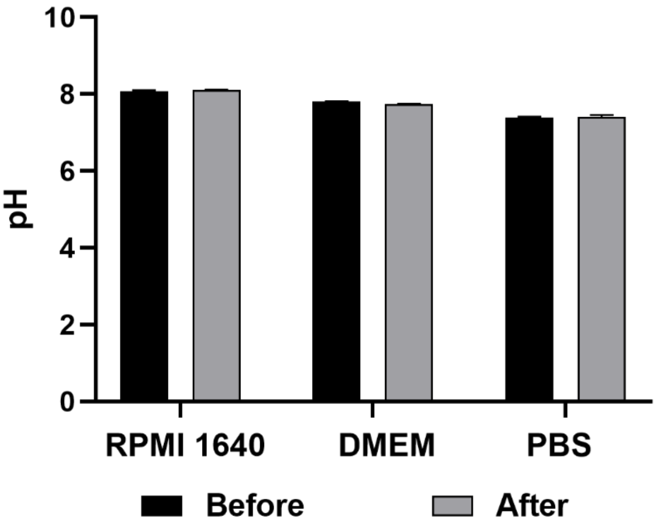

Fig. S3. Changes in pH of isotonic solution before and after impedance measurements. (Data are means ± SD, n=3).

Image of western blot:

To display proteins of different sizes on a single membrane, the membranes were cut prior to antibody hybridization. The images below are the original image with the edge of the membrane. Explain it in the method.

1. CyclinD1 and Actin.

**CyclinD1**	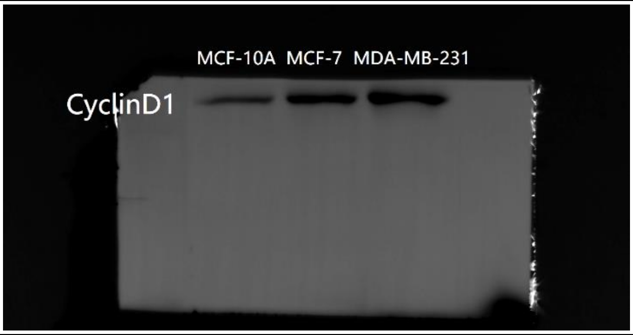
**Actin**	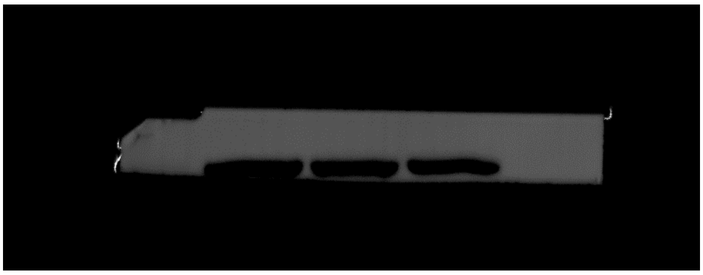

2. Ki67 and Actin

Due to the poor specificity of Ki67 antibody, the whole membrane was gray. In order to see more clearly, we used another contrast image from the device. Contrast was not artificially adjusted.

**Ki67**	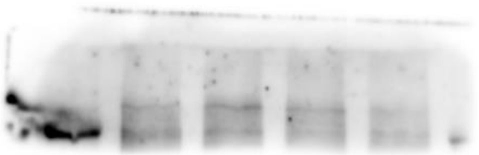
**Actin**	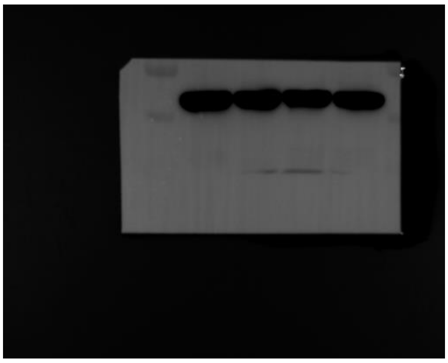

3. **LDHA, HIF and Actin**

<table>
<tr><td>LDHA</td><td>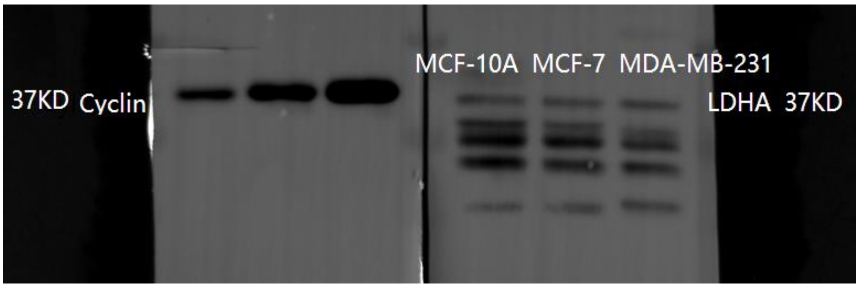
</td></tr>
<tr><td>HIF-1</td><td>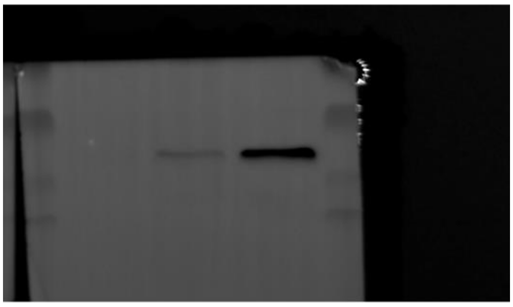</td></tr>
<tr><td>Actin</td><td>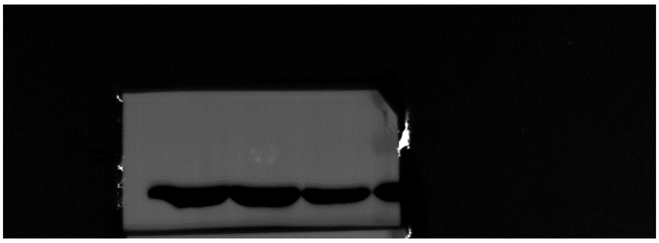</td></tr>
</table>

4. **NHE1 and Actin**

<table>
<tr><td>NHE1</td><td>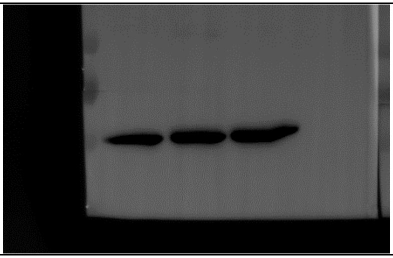</td></tr>
<tr><td>Actin</td><td>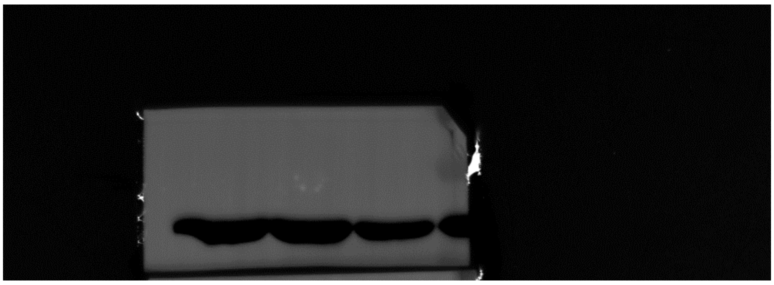</td></tr>
</table>
